# Role of the three-dimensional printing technology in complex laparoscopic renal surgery: a renal tumor in a horseshoe kidney

**DOI:** 10.1590/S1677-5538.IBJU.2019.0085

**Published:** 2019-12-17

**Authors:** Clàudia Mercader, Antoni Vilaseca, Javier Luis Moreno, Antonio López, Maria Carmen Sebastià, Carles Nicolau, Maria José Ribal, Luís Peri, Mertixell Costa, Antonio Alcaraz

**Affiliations:** 1 Department of Urology, Clinic Hospital of Barcelona, Barcelona, Spain; 2 Department of Radiology, Clinic Hospital of Barcelona, Barcelona, Spain

**Keywords:** Surgical Procedures, Operative, Laparoscopy, Kidney

## Abstract

**Purpose:**

To report our initial experience using a patient-specific 3D-printed renal tumor model for the surgical planning of a complex heminephrectomy in a horseshoe kidney.

**Materials and Methods:**

We selected a clinical case for a complex laparoscopic surgery consisting in a 53 year-old male presenting a local recurrence of a renal tumor in a horseshoe kidney with aberrant vascularisation previously treated with a laparoscopic partial nephrectomy. He is now proposed for a laparoscopic left heminephrectomy. Along with conventional imaging, a real-size 3D-printed renal model was used to plan de surgical approach. The perioperative experience of the surgical team was recorded.

**Results:**

The surgical team found the patient-specific 3D printed model useful for a better understanding of the anatomy and an easier surgical planning.

**Conclusion:**

The use of patient-specific 3D-printed renal models seem to be helpful for the surgical planning in complex renal tumors.

## INTRODUCTION

Renal cell carcinoma (RCC) represents 2%-3% of all malignancies in Western countries with a peak incidence between 60 and 70 years of age (1). Due to the widespread use of imaging techniques, more than 50% of RCC are nowadays diagnosed incidentally and smaller in size and at an earlier stage (2, 3). Because of this diagnostic shift and the introduction of laparoscopic surgery, the treatment paradigm of kidney cancer has shifted towards laparoscopic partial nephrectomy. In fact, laparoscopic surgery can offer faster patient recovery with similar postoperative complication rates and oncologic results as open surgery (4-6).

The estimated incidence of horseshoe kidneys in the general population is 0.15%-0.25% and incidence and prognosis of renal masses do not differ from those of the general population (7). However, horseshoe kidneys are associated with several anatomic abnormalities such as the presence of aberrant and accessory blood vessels and fusion or anatomical variants of the urinary tract (8). Together with the limited renal mobilization, any laparoscopic surgery becomes more challenging among these patients. Laparoscopic surgery in a horseshoe kidney is a highly complex procedure, where experience in laparoscopy and thorough surgical planning will be basic for surgical success (9-12).

The use of 3-dimensional (3D) images can contribute to a better understanding of the relationship between the vessels, urinary tract, kidney and tumor. The 3D reconstructed renovascular anatomy may help with planning the surgery in advance and ultimately improve oncological results and reduce complications. 3D printing technology has undergone an expansion in recent years and has yielded encouraging results in many fields from aeronautics to agriculture as well as clinical medicine. The use of 3D models can be very useful for planning surgery since it recreates the intraoperative scenario more precisely, providing an additional tool to understand the size, location, vascularization, relation with the collecting system and depth of a renal mass preoperatively (13-20).

As far as we know, we present the first case described in literature that uses a patient-specific 3D-printed renal model along with conventional imaging to plan the surgical approach in a highly complex salvage treatment with a heminephrectomy for a local recurrence of a renal tumor after a partial nephrectomy in a horseshoe kidney with aberrant vascularization.

## MATERIALS AND METHODS

### Clinical case

We present a 53 year-old Caucasian male with no allergies or other past medical history other than renal cancer.

A horseshoe kidney with a suspicious 4cm renal mass was incidentally found in 2012 during a routine abdominal ultrasound. A CT scan was performed showing a 4cm exophytic mass in the middle posterior left hemi-kidney. On June 2012 a laparoscopic partial nephrectomy was performed without perioperative complications. The pathology report revealed a clear cell renal cancer, Fuhrman II, pT1b tumor (5x4x3.5cm) with negative margins.

**Figure 1:**
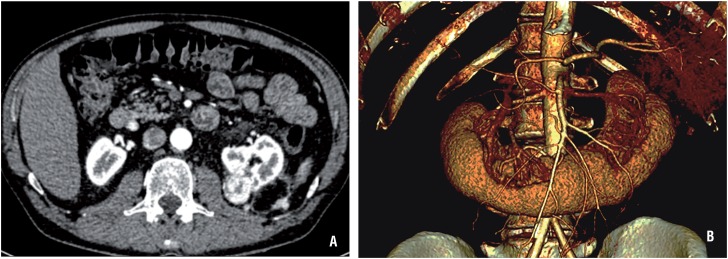
A) Angio-TC scan showing a 3.3 x 2.6 cm tumor in previous surgical bed, partly exophytic and reaching the renal sinus. Three solid nodules in the left posterior pararenal fascia of 0.9, 1.3 and 1.6 cm are also identified. There are identified two main renal arteries, one left and one right, and three accessory arteries originated in the anterior margin of the aorta, directed behind the renal isthmus, two to the left and one to the right. Besides, there are two left renal veins, the inferior with a retroaortic trajectory, and three right renal veins. B) Picture of the 3D reconstructed renovascular video images.

After four years without evidence of disease the patient presented with a 3 cm tumor in the surgical bed reaching the renal sinus. In addition, three solid nodules in the left posterior pararenal fascia of 0.9, 1.3 and 1.6cm were also identified. The angio CT-scan (Figure-1A) described one main renal artery and two accessory arteries originated in the anterior margin of the aorta and directed behind the renal isthmus. The inferior mesenteric artery crosses the isthmus near the accessory arteries. The vein anatomy consisted of two left renal veins (one inferior and retroaortic). The 3D reconstructed renovascular video images (in a 2D platform) were available (Figure-1B).

Discussed in a multidisciplinary team, the patient was offered a laparoscopic left heminephrectomy. Given that the patient had a horseshoe kidney with a complex vascular supply, it was of utmost importance to be aware of the anatomic relationships in order to prevent vascular lesions, such as the inferior mesenteric artery or the right renal inferior artery.

### 3D printing process

**Figure 2:**
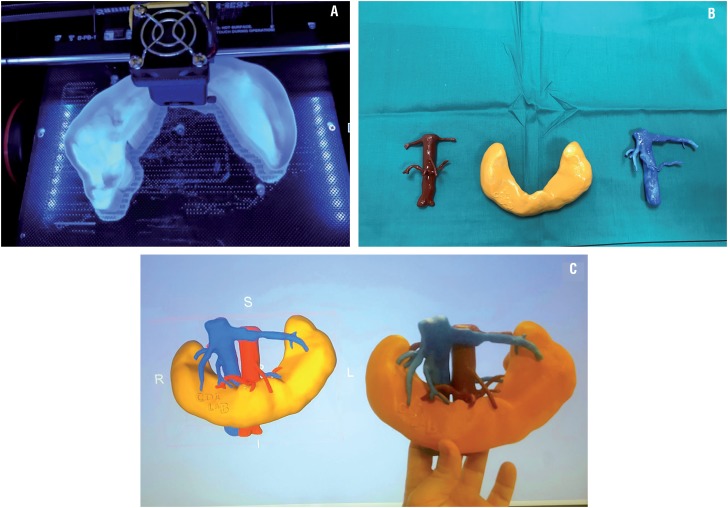
A) Printing process with the fused deposition modeling 3d printer of the horseshoe kidney piece in acrylonitrile butadiene styrene plastic that would posteriorly be painted. B) Each of the real size 3D-printed pieces after being painted, red for the arterial system, blue for the venous system and orange for the kidney. All three regions were dockable and detachable. C) The physical kidney models were compared to the 3D reconstruction and the patient radiology data to confirm spatial coherence.

Pre-operative images were acquired on a computerized angiotomography scan three weeks prior to surgery, including arterial, venous, and excretory phases. Images were imported and processed with a dedicated software platform used for 3D visualization, image segmentation and generation of stereolithography files. The kidney tissue, the arterial and the venous system were segmented as three separate anatomical regions of interest and converted to a separate object combined into a 3D virtual model (13). Each region was printed with a 3D fused deposition modelling printer (Beijing Tiertime Technology Co. Ltd.,) to full-scale size in acrylonitrile butadiene styrene plastic that was posteriorly painted using different combinations of rigid cyan and rigid magenta so that the arterial system remained red, the venous system blue and the kidney a collecting system orange-brown. The physical kidney models were compared to the 3D reconstruction and the patient radiology data to confirm spatial coherence. All three regions were dockable and detachable (Figure-2).

### Preoperative surgical planning

The CT angiography images together with the 3D reconstruction and the printed 3D demountable model were evaluated by the surgical team. The decision was to access from the upper pole to first identify and section the main left renal artery, and then the superior left vein. Later, in the caudal direction, the two accessory left arteries and the inferior retroaortic left lower vein would be sectioned. Once the vascular supply was controlled, the left heminephrectomy would be carefully carried out.

### Surgical procedure

**Figure 3:**
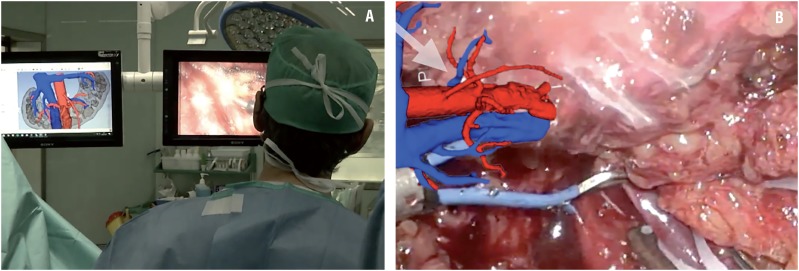
A) During the procedure, CT images and 3D reconstruction were available on the operating theater 2D screens as well as the patient-specific 3D-printed renal model. B) The 3D model faithfully reproduced the real anatomy of the patient and was useful for a better laparoscopic navigation and to confirm the identity of the vessels during the surgery.

During the procedure, CT scan images and the virtual 3D reconstruction were available in the operating room on 2D screens as well as the patient-specific 3D-printed renal model. We used 3D laparoscopic technology for the surgery (Figure-3A).

The patient was placed in right lateral decubitus. A left pararectal iterative 10mm incision for introduction of the optics was performed. Trocars were placed in the left iliac fossa, left flank and left hypochondrium. Lysis of multiple adhesions due to previous surgery was carried out and the upper left renal pole was released from the colon, spleen and jejunum. The main left renal vein and artery were identified and then sectioned between hem-o-loks^®^. Caudal dissection of the left hemikidney was carefully performed with the identification of the inferior mesenteric artery, which was preserved. The left ureter was identified and sectioned. The two left accessory arteries and retroaortic vein were identified and sectioned between hem-o--loks^®^ (Figure-3B). The limit of the isthmus was identified as there is a colour change due to the lack of perfusion. The isthmus was sectioned with scissors and the remaining right renal pole wound was closed with a barbed suture. The rest of the left kidney was resected, as was the left perirenal fat, including the perisplenic fat. The surgical sample was retrieved and referred for pathological study. The abdominal wall incisions were closed with absorbable stitches and skin was closed with staples.

The patient did not present any major complication during the postoperative period. The final pathology report evidenced a clear cell renal cell carcinoma ISUP 3, pT3a with negative margins.

## RESULTS

The head surgeon claimed that the use of the patient-specific 3D-printed model was helpful as a complement to conventional imaging for a better understanding of the disposition of the vascularization of the kidney, which allowed a better pre-surgical mental planning and intraoperative anticipation during the procedure. The second surgeon admitted that the patient-specific 3D-printed model offered a reliable picture of the anatomy allowing a better understanding of the surgical approach which helped them to remain synchronized with the main surgeon for better anticipation and assistance performance. They all agreed that the model faithfully reproduced the real anatomy of the patient and that it was useful to identify and confirm the identity of the vessels when dissected and sectioned. Finally, the resident who assisted the surgery agreed that the 3D-printed model helped them to preoperatively understand the tridimensional anatomical structures and the surgical plan, thus, they could easily follow the live surgery in a much more understandable way.

## DISCUSSION

The laparoscopic approach to treat renal cancer in a horseshoe kidney implies greater complexity due to the presence of aberrant vessels, abnormal kidney position, the presence of the isthmus, and other associated abnormalities (4-6). Therefore, detailed preoperative radiological evaluation of the anatomy and careful surgical planning are essential. In this era of technological development, the use of 3D laparoscopy has been incorporated into the surgical routine of many hospitals. However, the use of 3D imaging studies such as printed models or the use of augmented reality for surgical planning is not yet widespread in daily care activity. The potential role of patient-specific 3D-printed models in urology has been scarcely studied to this point and most of the developments have been used for partial nephrectomies. It has been previously reported that patient-specific 3D-printed renal tumor models contribute to an improved comprehension of the tumor anatomy and the proposed surgical planning for trainees, patients and their family members, and are also useful in surgical training simulators for residents and young urologists (14, 17, 19).

Until now, the main benefit of patient-specific 3D-printed renal tumor models is their usefulness in surgical planning. For instance, Zhang et al., created 3D-printed renal tumor models of ten patients developed for planning laparoscopic partial nephrectomy procedures that were evaluated preoperatively by five experienced laparoscopic urologists with a questionnaire about the overall usefulness, realism and whether the models were a useful tool in surgical planning and training. All participating urologists agreed and advocated that it was useful for surgical planning and potential training of demanding procedures, when combined with 2D data. Postoperatively, the two operating urologists claimed that intraoperative navigation with those models was helpful in presenting tumor details, its relationship with adjacent structures, resection range, and prevention of key structures injuries, especially for hilar tumors (20). These results are consistent with those found by Wake et al., who retrospectively selected ten complex renal mass cases and generated their respective 3D-printed models. Three experienced uro-oncology surgeons evaluated and presumed the preoperative approaches regarding partial or radical nephrectomy, open or laparoscopic approach, transperitoneal or retroperitoneal approach and clamping with and without the 3D model. There was a change in planned approach with the 3D-printed model for all cases with the largest impact seen regarding decisions on transperitoneal or retroperitoneal approach (30-40%) and clamping (40-50%). The concordance between the actual surgical approach and preoperative surgical planning improved with the use of the 3D models. More than that, all the surgeons reported that the 3D printed model helped with comprehension of anatomy with regards to decisions on surgical approach and increased their confidence that they correctly planned the procedure, which indicates that even experienced urologists may potentially benefit from the 3D printed models for planning complex surgeries(18). Patient-specific 3D renal models may also be useful for an easier tumor margin detection. Komai et al. designed 3D-printed, highly complex, renal tumor models of ten patients so that the tumor and its margin could also be removed, which helped visualize both the pre and post tumor resection kidney status. They noted that the patient-specific 3D printed kidney model allowed their discussions to be more detailed than they would have been with either cross-sectional 2D or 3D reconstructed images, especially to identify definite tumor feeders in order to perform off-clamp partial nephrectomies. During the surgery, they confirmed that the 3D printed kidney model was consistent with the intraoperative appearance of the tumor surface before resection, the way the feeder travelled and the status of the post-resection remnant kidney, and that it significantly shortened the duration of intraoperative ultrasound. The models allowed surgeons to preoperatively picture the real intraoperative scenario facilitating minimally invasive off-clamp laparoscopic partial nephrectomy (15). Finally, patient-specific 3D renal models can be also useful for surgical training prior to the surgery as described by von Rundstedt et al., who generated patient-specific models for ten patients with complex tumor anatomy that were used to perform a preoperative rehearsal of the robotic-assisted partial nephrectomy before the actual procedure, all performed by the same surgeon. They realized that the pre-surgical rehearsal on the model significantly altered their approach to the actual tumour in several cases, thus improving the resection strategy in complex cases (16).

All these studies seem to indicate that the real 3D-printed models may be useful for better surgical planning since they provide a consistent picture of the intraoperative scenario and facilitate the comprehension of anatomy. However, these are feasibility studies limited by the small sample size and its design, such that a large amount of evidence cannot be provided. It is also difficult to objectively evaluate the subjective perception of different surgeons facing distinct and unique clinical cases. Further comparative studies are required to evaluate the potential benefits of 3D model-based surgery for preoperative surgical planning and its role in enhancing surgical performance in complex renal surgery.

## CONCLUSIONS

The use of patient-specific 3D-printed renal models provides a better reproduction of the anatomic structures that can be useful to plan the surgical strategy in highly complex tumors, facilitating the comprehension of the spacial anatomy and a better identification of risk areas of injuries, probably leading to an enhanced final surgical performance. Further studies are needed to confirm this opinion.

## ABBREVIATIONS

RCC: Renal cell carcinoma
3D: 3-dimensional
CT scan: computed tomography scan
